# Community structure of environmental microorganisms associated with COVID-19 affected patients

**DOI:** 10.1007/s10453-021-09708-5

**Published:** 2021-05-04

**Authors:** Weihua Zhang, Guoxin Mo, Jie Yang, Xingshuo Hu, Hujie Huang, Jing Zhu, Pei Zhang, Han Xia, Lixin Xie

**Affiliations:** 1 College of Pulmonary and Critical Care Medicine, Chinese PLA General Hospital, Beijing, 100853 China; 2 Hugobiotech Co. Ltd, Beijing, 100000 China; 3 Shanghai Institute of Optics and Fine Mechanics, Chinese Academy of Sciences, Shanghai, 201800 China; 4 Shanghai Lasensor Photoelectric Technology Co., Ltd, Shanghai, 201800 China

**Keywords:** COVID-19, Aerosol, Hospital microbiome, Environmental microbiology, Metagenomic next-generation sequencing (mNGS)

## Abstract

To clarify the characteristics and distribution of hospital environmental microbiome associated with confirmed COVID-19 patients. Environmental samples with varying degrees of contamination which were associated with confirmed COVID-19 patients were collected, including 13 aerosol samples collected near eight patients in different wards, five swabs from one patient’s skin and his personal belongings, and two swabs from the surface of positive pressure respiratory protective hood and the face shield from a physician who had close contact with one patient. Metagenomic next-generation sequencing (mNGS) was used to analyze the composition of the microbiome. One of the aerosol samples (near patient 4) was detected positive for COVID-19, and others were all negative. The environmental samples collected in different wards possessed protean compositions and community structures, the dominant genera including *Pseudomonas*, *Corynebacterium*, *Neisseria*, *Staphylococcus*, *Acinetobacter*, and *Cutibacterium*. Top 10 of genera accounted for more than 76.72%. Genera abundance and proportion of human microbes and pathogens radiated outward from the patient, while the percentage of environmental microbes increased. The abundance of the pathogenic microorganism of medical supplies is significantly higher than other surface samples. The microbial compositions of the aerosol collected samples nearby the patients were mostly similar to those from the surfaces of the patient's skin and personal belongings, but the abundance varied greatly. The positive rate of COVID-19 RNA detected from aerosol around patients in general wards was quite low. The ward environment was predominantly inhabited by species closely related to admitted patients. The spread of hospital microorganisms via aerosol was influenced by the patients’ activity.

**Supplementary Information:**

The online version contains supplementary material available at 10.1007/s10453-021-09708-5.

## Introduction

Since the COVID-19 outbreak at the end of 2019 in Wuhan, China, the public healthcare systems borne unprecedented burdens globally. So far, more than 4.2 million confirmed cases were reported all over the world (Bray et al., 2020; WHO, 2020), and the number continues rising under the rapid spread. The causative pathogen is highly infectious and can cause severe pneumonia (Chan et al., 2020). Current studies showed that COVID-19 had high aerosol and surface stability (Doremalen, 2020). The virus remains viable and infectious in aerosol for hours. The small particles with COVID-19 contents may travel in indoor environments, covering distances up to 10 m starting from the emission sources (Morawska & Cao, 2020). Also, close contact with COVID-19 contaminated surfaces is considered to be one of the possible routes of transmission (Paules et al., 2020). So the effect of the patient to the surrounding environment was especially significant to the control and prevention of COVID-19.

In recent years, hospitals were regarded as pathogens’ ecosystems. The built environment of hospitals was more like a waiting room for these potentially harmful bacteria until suitable conditions emerged (Arnold, 2014). Several studies showed the widespread distribution of COVID-19 on the surfaces and in the air of ICU and isolated wards (Chia et al., 2020; Jiang et al., 2020; Wu et al., 2020). However, there were some researchers failed to obtain the virus from the environmental samples (Colaneri et al., 2020; Li et al., 2020; Wang et al., 2020). The reason of these inconsistent results remains unknown. In this study, metagenomic next-generation sequencing (mNGS) was utilized to detect the microbiome of the aerosol samples and swabs collected from the surrounding environment of COVID-19 patients, with the aim to analyze the abundance, identity, and concentration of the microbial composition, and to provide important information for developing hospital infection prevention and control measures.

## Method

### The experiment design and the sampling plan

**Fig. 1:**
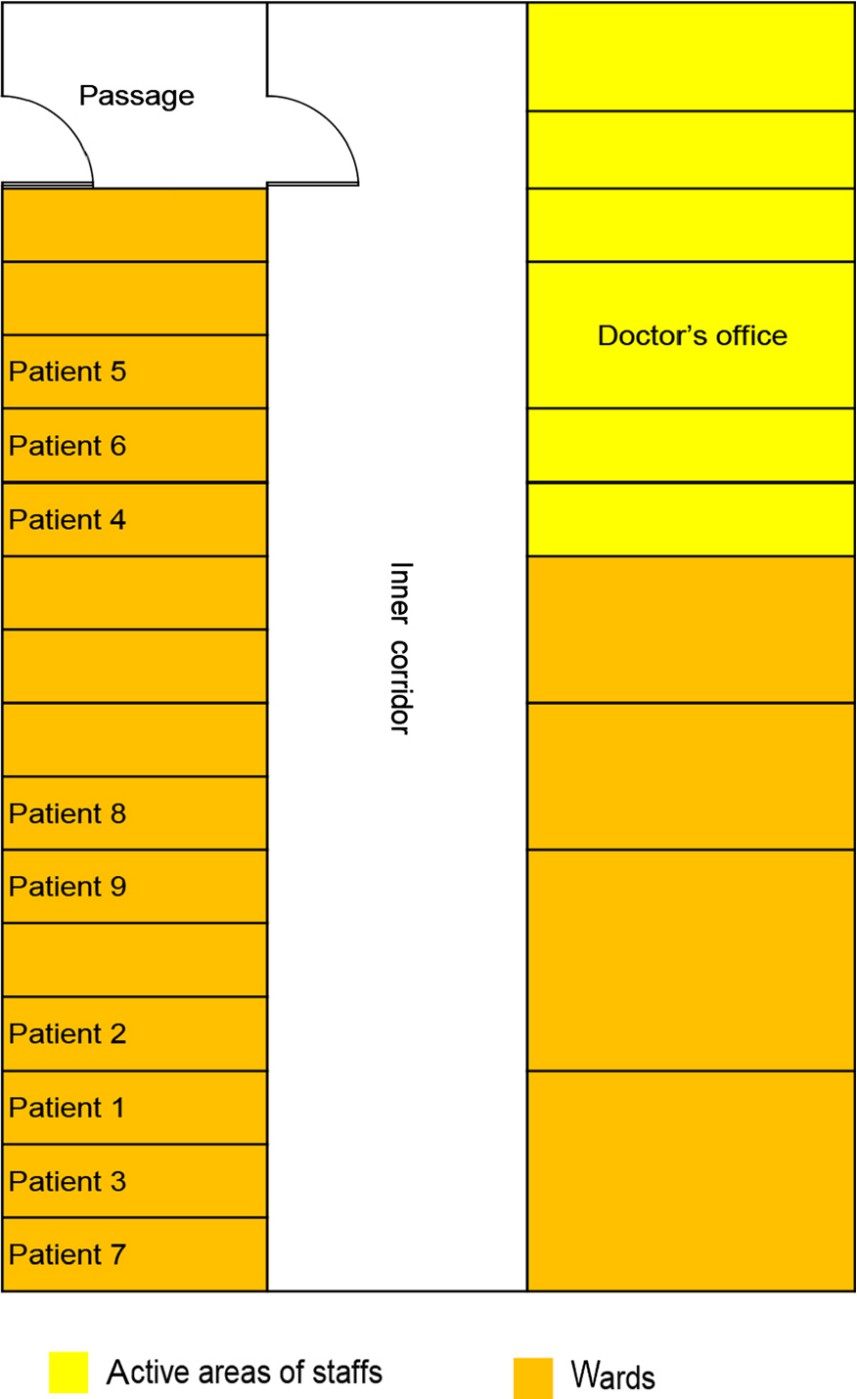
A detailed map of the distribution of the wards/ICU with COVID-19 patients

Five female and four male patients were included in this investigation, with ages ranging from 20 to 68 years old. They were all diagnosed positive for COVID-19 clinically by a PCR-based method, all experiencing mild symptoms at the time of sampling in our hospital, except patient 5 who developed severe pneumonia symptoms. All the COVID-19 patients were isolated in single wards, with very little human activity. The medical staffs entered the wards for rounds once a day, wearing personal protect equipment (PPE) with no skin exposed. The patients were not allowed to exit the room. The airtight wards were not disinfected during the illness. A detailed map of the distribution of the wards is shown in Fig. 1. Due to the constrain of COVID-19 outbreak, general wards were turned into temporary ICUs by supplemented with necessary equipment.

We collected 13 bioaerosol samples and 7 swab samples from February 28, 2020 to March 28, 2020. One aerosol sample collected from doctor’s office (uncontaminated regions) was used as control, while the other samples were all collected around COVID-19 patients and corresponding environment in common wards and RICU. Sampling details are described in Table 1.

**Table 1:** Sampling position and time points in relation to patient’s clinical information

Sample no.	Specimen type	Patient no.	sex	ages	Days after symptom onset	Symptoms at the time of sampling	Room type	mNGS testing for COVID-19	Samples collected points
OA1	Aerosol							Negative	Air of the doctor’s office
AA1	Aerosol	1	Female	67	29d	No fever and cough	General ward	Negative	On the table next to the patient (< 0.5 m from patient)
BA1	Aerosol	2	Female	78	48d	Unconscious	ICU	Negative	In the doorway
BA2	Aerosol								On the table next to the patient (< 0.5 m from patient)
CA1	Aerosol	3	Male	27	3d	Cough, suffocated	General ward	Negative	Distance from patient < 1 m
CA2	Aerosol								Distance from patient < 3 m
CA3	Aerosol								Distance from patient < 5 m
DA1	Aerosol	4	Female	48	8d	Cough	General ward	Positive	On the table next to the patient (< 0.5 m from patient)
DS1	Swab				13d	Cough		Negative	Hand
DS2	Swab								Cellphone
DS3	Swab								Pillow towel
DS4	Swab								Bed surface
DS5	Swab								The corners of the mouth and cheeks
DA2	Aerosol								On the table next to the patient (< 0.5 m from patient)
ES1	Swab	5	Female	68	29d	Fever, Cough, suffocated	ICU	Negative	Positive pressure respiratory protective hood from the physician performed bronchoscope for the patient
ES2	Swab								Face shield from the physician performed bronchoscope for the patient
FA1	Aerosol	6	Female	20	16d	Throat discomfort	General ward	Negative	On the table next to the patient (< 0.5 m from patient)
GA1	Aerosol	7	Male	58	53d	Multiple lesions in lungs	General ward	Negative	On the table next to the patient (< 0.5 m from patient)
HA1	Aerosol	8	Male	42	28d	Cough	General ward	Negative	On the table next to the patient (< 0.5 m from patient)

The first letter ‘A’–‘I’ of each No. represent the patient 1–9, the second letter of each No. ‘S’ means the swab sample, and ‘A’ means the aerosol sample, the “OA1” means the aerosol sample from the office of doctor

Aerosol samples were collected at a flow rate of 400 L/min for 30 min by an aerosol particle liquid concentrator LS-400 (Shanghai Lasensor Optoelectronic Tech). The particulate matter collected from the air was blown into a strain preservation tube with 3 ml collection fluid buffer AVL for filtering and lysis of microorganisms.

In general, aerosol samples were collected at 0.5 m, 1 m, 3 m, and 5 m from the patients. PPE from a physician who had just performed suctioning for a patient with COVID-19 was sampled using sterile pre-moistened swabs. Surfaces of skin and personal items from a COVID-19 patient were also collected by using sterile pre-moistened swabs. All samples were stored at − 80 °C.

The collection tubes were transported to Hugobiotech Co., Ltd (Beijing, China) on dry ice within 48 h. PACEseq mNGS tests were performed immediately, and bioinformatics analysis was carried out. To ensure the prospectiveness of this study, mNGS technicians had no access to patients’ clinical data.

### Nucleic acid extraction, library preparation, and mNGS sequencing

DNA was extracted and purified from 200 μl aerosol samples or swabs according to the instruction of DNA Mini kit DP316 (Tiangen). RNA was extracted by QIAamp Viral RNA Mini Kit 52,906 (Qiagen), following a reverse transcription using SMART MMLV Reverse Transcriptase kit 639,524 (Takara). The concentration and quality of DNA & cDNA were checked using Qubit (Thermo Fisher) and agarose gel electrophoresis. Sterile water was set as negative controls for each reaction.

The DNA and RNA libraries were constructed using QIAseq™ Ultra low Input Library Kit (Qiagen) according to the handbook. The concentration and quality of libraries were measured using Qubit and agarose gel electrophoresis. Qualified libraries with different barcode labelings were pooled together, followed by amplification and enrichment, and then sequenced on an MiniSeq platform (Illumina).

### Bioinformatics pipeline

Clean reads were generated by removing low-quality and short reads (length < 50 bp) from raw sequence data, followed by computational subtraction of human host sequences mapped to the human reference genome (hg19) using SNAP. The remaining data were further classified by Burrows-Wheeler alignment to four Microbial Genome Databases (Refseq), downloaded from National Center Biotechnology Information (ftp://ftp.ncbi.nlm.nih.gov/genomes/), containing genetic information of 11,910 bacteria, 7103 viruses, 1046 fungi, and 305 parasites associated with human diseases.

### Data analysis

The microbiome diversity of the environmental samples was assessed, with the microbial components categorized into three categories: (1) environmental microbes, microbes abundant in the environment (2) pathogens (3) human microbes, the microbial organisms inhabited in the human body, such as in oral cavity, gut, skin, and vagina. The classification in detail is shown in Table S1.

## Results

### Sampling mode and demographic characteristics

Aerosol samples OA1, AA1, BA1, and BA2, from the doctor’s office, doorway of a COVID-19 patient ward, and around two COVID-19 patients separately, were used to assess succession characteristics of microflora in different habitats. Samples CA1, CA2, and CA3, from the same patient (patient 3), were used to assess the relationship between microflora distribution and distance from patients. Surface swabs from patient 4 were analyzed since the aerosol DA1 around him was detected positive for COVID-19.

Only two samples (CA1 and DA1) collected from the area around patient were less than 10 days after symptoms occurred, while the rest samples from patients were more than 10 days, the maximum time was 72 days (Table 1).

DA1, an aerosol sample of patient 4 collected on the 8th day after symptoms occurred, was detected positive for COVID-19 with two specific sequences. However, the corresponding swabs for her skin and personal belonging were all negative for COVID-19. All other aerosol and swab samples of the rest patients were negative for COVID-19.

### Taxonomic diversity of samples in medical environments of COVID-19 patients

**Fig. 2:**
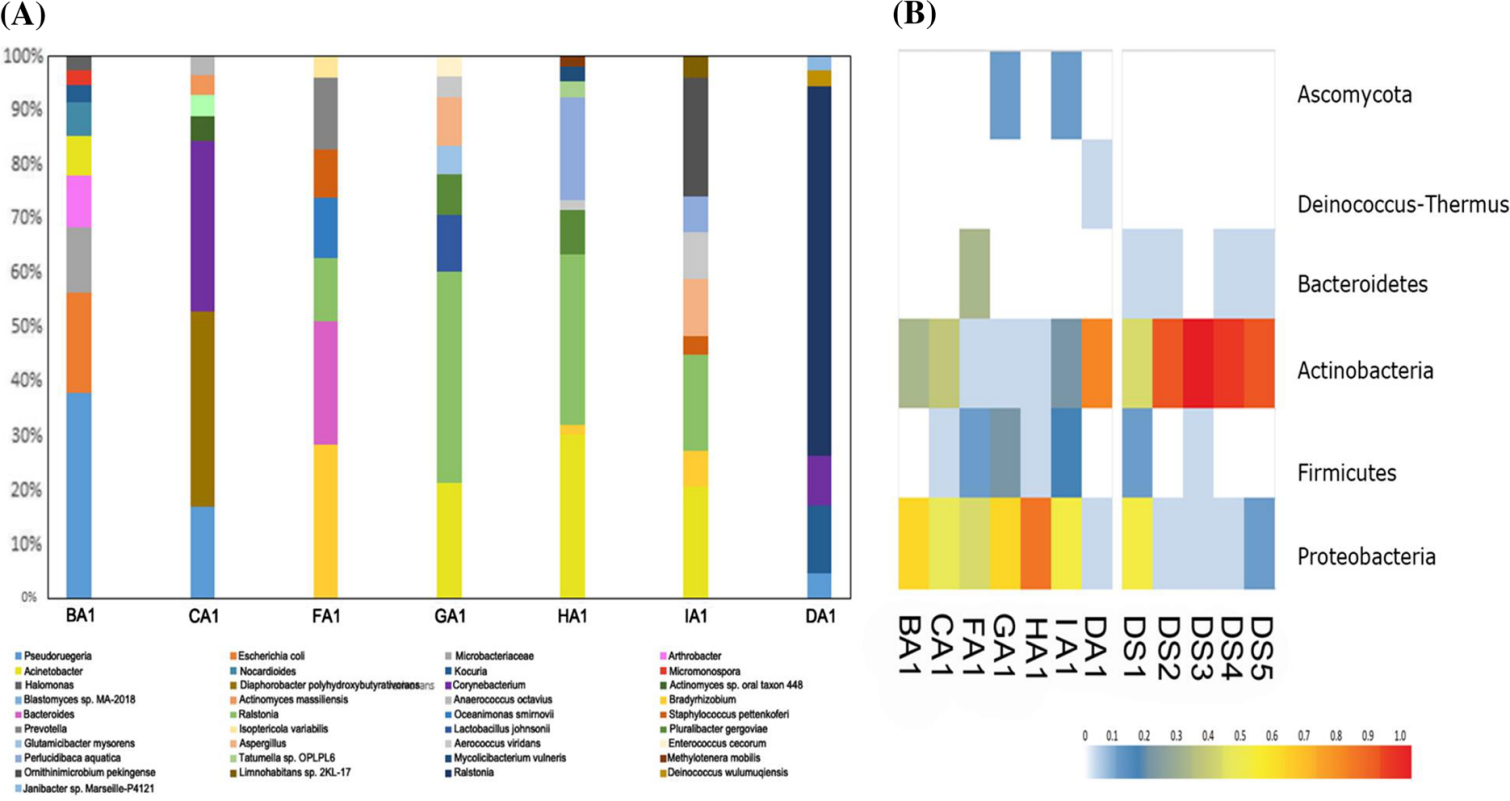
**a** Genera composition of environmental samples from COVID-19 patients. **b** Phylum abundance of environmental samples from COVID-19 patients

The dominant microorganisms (Top 10) revealed by mNGS are shown in Fig. 2a for the diversity of genera, and in Fig. 2b for the diversity of phylum. Top 10 took for over 76.72%, with the highest proportion at 99.76%. The percentage of dominant species are shown in Table S2.

Genera of bacteria, fungi, viruses were identified by mNGS from aerosol samples. The diversity of genera exhibited inconsistency with every sample (Fig. 2a), whereas the components in phylum revealed consistency (Fig. 2b). Proteobacteria was found with the highest richness in aerosols, followed by Actinobacteria and Firmicutes, whereas Actinobacteria were the most abundant in swabs, and it was not significant for the abundance of Bacteroidetes, Actinobacteria, or Firmicutes.

### Distribution characteristics of samples in medical environments of COVID-19 patients

**Fig. 3:**
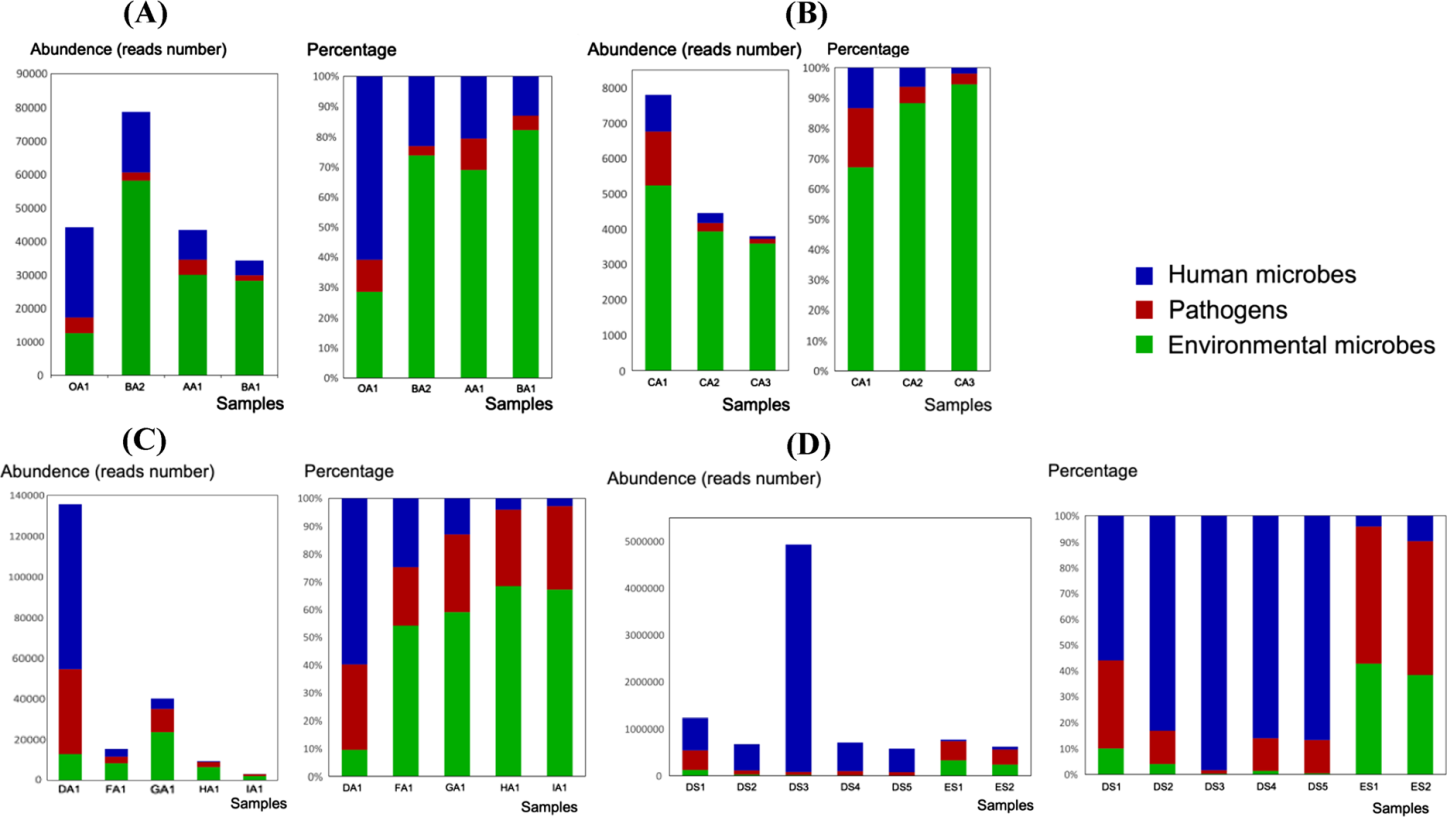
Community structure of environmental samples associated with COVID-19. The abundance (reads numbers) and proportion of different kinds of microorganisms at different levels (**a**) from the clean area to the contaminated area. **b** At the distance of 1 m, 3 m, and 5 m from patients. **c** Aerosol samples of different patients affected with COVID-19. **d** Swabs from the patient’s hand, cellphone, pillow towel, bed, cheek, medical staff’s positive pressure respiratory protective hood, and face shield

To elucidate characteristics of microflora succession in different habitats, four specimens from the doctor office (non-contaminated area), the doorway of the hospital ward (semi-contaminated area), and two tables by the two patients beds (contaminated area) were obtained, and the result is shown in Fig. 3a. The microbial community structure of semi-contaminated and contaminated areas appeared quite similar. The abundance of human microbes from the clean area was much higher than the other areas of the ward, reaching 60% of the total microorganisms. On the other hands, environmental microbes were the dominant microorganisms in ward, reaching 70–80%. The amount and percentage of pathogens from doorway (semi-contaminated area) shared the same pattern with that of the doctor’s office (non-contaminated area). Environmental bacteria *Pseudoruegeria sabulilitoris*, with a large share (30–65%) of microbiome in the patient’s ward, only made up for just 6.86% in sample collected from the doctor’s office. *Moraxella osloensis*, *Kocuria rosea*, and *Acinetobacter* are the major components of pathogens from the aerosol.

To assess microflora distribution at different distances from the patient, air samples next to a patient at the distance of 1 m, 3 m, and 5 m were collected (Fig. 3b). Environmental bacterial *Diaphorobacter polyhydroxybutyrativorans*, *Pseudomonas* accounted for more than 40% of the aerosol and were the dominant bacteria of the airborne microbes. *Corynebacterium propinquum*, *Corynebacterium aurimucosum*, and *Kocuria marina* are the major pathogens detected. The abundance of pathogens and human microbes decreased remarkably along with the distance, while the proportion of environmental microbes increased with distance.

We evaluated the composition of microbes of aerosols from COVID-19 patients. GA1, HA1, and IA1 were collected around different patients at the same day, and DA1 and FA1 were collected earlier. Environmental bacteria *Bradyrhizobium sp.*, *Ralstonia mannitolilytica*, *Acinetobacter*, and *Ornithinimicrobium pekingense* are the dominant bacteria, accounted for more than 30% of total microbes. The main pathogens were *Acinetobacter*, *Aerococcus viridans*, and *Staphylococcus pettenkoferi*. The total microorganisms’ abundance was significantly different in different samples. However, environmental microbes occupied a considerable proportion of microbiome around the area of COVID-19 patients in the hospital ecosystem. The ratio of pathogens was relatively stable, which varied between 20 and 30%.

Surface swabs of exposure objects associated with patients and their physicians are shown in Fig. 3d. Environmental microorganisms *Pseudocitrobacter faecalis* and *Pantoea eucrina* accounted for 37.59% and 24.66% of hand surface microbes of a patient, respectively. For surface swabs of the patient’s cellphone, cheek, towel, and bed, the environmental microorganisms *Cutibacterium acnes* accounted for a considerable proportion of microbiome over 60%, followed by *Propionibacterium humerusii*, 6–30%.

Pathogens *Enterococcus cecorum* and *Burkholderia multivorans* were the dominant bacteria of PPE surface microbial community, accounted for 12–25% in both swabs. Swab analysis of the surfaces of patients and their personal belongings revealed similar nucleic components with the majority of species identified from the human body and accompanied by different pathogens and a small number of environmental microbes. Two swabs analysis of PPE showed that pathogens and environmental microbes took up a great proportion, while the proportion of human microbes decreased accordingly.

## Discussion

The hospital wards of COVID-19 patients are new emergency and special circumstance. This study provided the first real-world investigation of ward environmental microbiome associated with confirmed COVID-19 patients by mNGS. We investigated the community structure characteristics of the special ecosystem, compared the environmental samples at different distances and different levels of pollution, and evaluated influence factors.

Aerosol transmission of COVID-19 has been suggested as an additional important pathway from clinic observations in confined spaces (WHO, 2020). In different sites of hospitals, COVID-19 positive air samples were detected, and the highest concentration was observed inside patient toilet room without ventilation (Chan et al., 2020; Zhu et al., 2020). Some studies found the concentrations of COVID-19 with a widespread distribution on the surfaces and in the air of the ICU and isolated wards (Chia et al., 2020; Jiang et al., 2020; Wu et al., 2020). But there are also some researches failed to obtain the virus from the environmental surfaces of related stuffs (Colaneri et al., 2020; Li et al., 2020; Wang et al., 2020). The viral detection rates of different patients vary considerably and were higher from environmental surfaces with more contact with the patients (Ryu et al., 2020). In this study, the sample DA1 was detected positive for COVID-19. Our results of aerosol samples also proved that viruses were present in the air of an isolation ward with confirmed patient with mild symptoms.

In this study, we found a very low detection rate of COVID-19 by mNGS in the aerosol and swab samples, which was consistent with other studies which also detected COVID-19 from aerosol samples at a very low rate (Chia et al., 2020; Li et al., 2020; Wang et al., 2020). In this study, the human activity of patients in isolated wards was quite low, and most of the patients only experienced mild symptoms of COVID-19, which might impact the detection rate. Further research would be needed to determine the influence of the air sampler, the flow rate, and the duration of aerosol sampling.

The microbiome of hospital environment is highly affected by the presence of human beings. Previous studies found that *Cutibacterium* spp. was the most frequent and abundant bacterial genera in operating tables and operating beds, while *Staphylococcus* spp. was the main contaminant of the floors (Comar et al., 2019). *Staphylococcus*, *Streptococcus*, *Corynebacterium*, and *Acinetobacter* were considered as the dominant genera of hospital (Lax et al., 2017). However, *Acinetobacter* and *Pseudomonas* were described as the largest proportion on hospital ecosystem in other studies (Rampelotto et al. 2019). The microbial communities colonizing in each region of different hospitals vary a lot. In this study, although *Acinetobacter* and *Pseudomonas* took up larger proportion, the dominant species in each environmental sample were different. Not only *Acinetobacter* and *Pseudomonas*, but also *Ralstonia*, *Enterococcus*, and *Bradyrhizobium* were detected as the dominant species of environmental microflora in different samples (Fig. 2a). It revealed that the structure and composition of the microbial community were heterogeneous within different patients in such a closed ward. It developed into an independent community, very similar to the patient's microbiota such as skin and belongings. So, the built of isolated wards could insulate the patients from each other and was effective for the prevention of COVID-19. Top 10 of genera took for over 76.72%, with the highest proportion 99.76%, which was much higher than 64.4% described in previous study. This might be caused by the accumulation of dominant strains during the quarantine time of the patients. The detailed information about the top 10 of all species and their percentage is shown in Table S2.

It was known that respiratory viral infections could alter the microbiome of body, such as pulmonary infections by influenza and respiratory syncytial virus (Deriu et al., 2016; Groves et al., 2018; Yildiz et al., 2018). Zuo et al*.* found gut microbiome was disturbed in patients with COVID-19 (Zuo et al., 2020). We also found many pathogens in aerosol and swab samples, some of which accounted for a large proportion. For example, the *Enterococcus cecorum* is uncommon in the air environment, but was detected as the main pathogen of DA2 (an air sample < 0.5 m from patient 4) and ES1 (a swab sample of positive pressure respiratory protective hood from the physician performed bronchoscope for the patient). The obtained pathogens were more likely from the patients with COVID-19. Though we did not detect the microbiome directly from body samples from patients, the specific distribution of pathogens with large proportions might indicate the disturbance of microbiome of patients with COVID-19.

We investigated the distribution of microbes in COVID-19 patients’ wards. In this hospital environment with extremely little interference from external environment, the microorganisms showed regular changes along the distance from the patient (Fig. 3b). The pathogenic microorganisms and human microorganisms radiated from the sickbed, with their abundance and proportion decreased, while the proportion of environmental bacteria increased. With the decrease in human activity, the pathogens and human microorganisms that closely related to human activities also decreased. This also meant that the community structure of the aerosols we detected perhaps was the result of the antagonistic effects of patients’ activities on the existing environment. Unfortunately, we did not detect any COVID-19 in these three samples, which made it impossible to determine the relationship between human activity and the virus distribution of environment. The PPE surface swabs which possessed high pathogen concentrations suggested that repeated contacts with patient's medical supplies were a potential approach to spread pathogens. Aerosol samples from doctors' offices showed higher levels of human microorganisms, possibly due to the activity of doctors and nurses bringing in the human microorganisms.

The results of the study had certain limitations. First of all, the common effect of the carbon dioxide concentration, temperature, and humidity to the environmental microbial abundance was not considered in our study, which might affect the deep understanding of the hospital microbiome. Inadequate samples were also an important drawback of this study. Limited by the number of patients and the duration of the disease, the insufficient sample content and the incomplete sampling method determined the limitations for the exploration of the hospital environmental microbes, which would also need more investigations in the future researches.

In general, the positive rate of COVID-19 RNA detection from aerosol around patients in general wards was quietly low. The air microorganisms in the strictly isolated wards mainly came from patients and the environment. The structure and composition of microbes community of different wards’ environment associated with COVID-19 patient were different. The spread of microorganisms of aerosol was influenced by the distance from patients.

## Supplementary Information

Below is the link to the electronic supplementary material.

Supplementary file1 (XLSX 19 kb)

## Data Availability

We declared that materials described in the manuscript, including all relevant raw data, will be freely available to any scientist wishing to use them for non-commercial purposes, without breaching participant confidentiality.
